# Modulation of extracellular *Penicillium expansum*-driven acidification by *Papiliotrema terrestris* affects biosynthesis of patulin and has a possible role in biocontrol activity

**DOI:** 10.3389/fmicb.2022.973670

**Published:** 2022-08-01

**Authors:** Davide Palmieri, Cecilia Miccoli, Ivan Notardonato, Pasquale Avino, Giuseppe Lima, Filippo De Curtis, Giuseppe Ianiri, Raffaello Castoria

**Affiliations:** ^1^Department of Agricultural, Environmental and Food Sciences, University of Molise, Campobasso, Italy; ^2^Department of Agricultural and Forestry Sciences, University of Tuscia, Viterbo, Italy

**Keywords:** *Papiliotrema terrestris*, patulin, *Penicillium expansum*, pH, mechanisms of biocontrol

## Abstract

The active regulation of extracellular pH is critical for the virulence of fungal pathogens. *Penicillium expansum* is the causal agent of green-blue mold on stored pome fruits and during its infection process acidifies the host tissues by secreting organic acids. *P. expansum* is also the main producer of patulin (PAT), a mycotoxin found in pome fruit-based products and that represents a serious health hazard for its potential carcinogenicity. While it is known that PAT biosynthesis in *P. expansum* is regulated by nutritional factors such as carbon and nitrogen and by the pH, the mechanistic effects of biocontrol on PAT production by *P. expansum* are not known. In this work, we assessed how optimal and suboptimal concentrations of the biocontrol agent (BCA) *Papiliotrema terrestris* LS28 affect both extracellular pH and PAT biosynthesis in *P. expansum*. In wounded apples, the optimal and suboptimal concentrations of the BCA provided almost complete and partial protection from *P. expansum* infection, respectively, and reduced PAT contamination in both cases. However, the suboptimal concentration of the BCA increased the specific mycotoxigenic activity by *P. expansum*. *In vitro*, the rate of PAT biosynthesis was strictly related to the extracellular pH, with the highest amount of PAT detected in the pH range 4–7, whereas only traces were detectable at pH 3. Moreover, both *in vitro* and in apple wounds the BCA counteracted the extracellular *P. expansum*-driven acidification maintaining extracellular pH around 4, which is within the pH range that is optimal for PAT biosynthesis. Conversely, in the absence of LS28 the pathogen-driven acidification led to rapidly achieving acidic pH values (<3) that lie outside of the optimal pH range for PAT biosynthesis. Taken together, these results suggest that pH modulation by LS28 is important to counteract the host tissue acidification and, therefore, the virulence of *P. expansum*. On the other hand, the buffering of *P. expansum*-driven acidification provided by the BCA increases the specific rate of PAT biosynthesis through the extension of the time interval at which the pH value lies within the optimal range for PAT biosynthesis. Nevertheless, the antagonistic effect provided by the BCA greatly reduced the total amount of PAT.

## Introduction

The modulation of extracellular pH plays a fundamental role during the attack on host plant tissues by many fungal pathogens (Prusky et al., 2001; Fernandes et al., 2017; Vylkova, 2017). For some of them the molecular mechanisms of pH sensing and adaptation have been elucidated. A key role is played by the Pac/Rim alkaline response pathway, which regulates the expression of genes involved in the adaptation to environmental pH (Antonio et al., 2010; Selvig and Alspaugh, 2011; Franco-Frías et al., 2014; Ost et al., 2015; Wu et al., 2016). On the other hand, fungi can actively modify the environmental pH by secreting acids or alkali. In the majority of necrotrophic phytopathogens, acidification of the extracellular environment is achieved through the secretion of acidic compounds such as butyrate, oxalate, malate, citrate, gluconate, and succinate, by removing ammonium ions from ammonium sulfate salt, and/or by excreting hydrogen (H^+^) ion as a byproduct of ammonium (NH^4+^) assimilation (Fernandes et al., 2017; Vylkova, 2017).

*Penicillium expansum* is an important post-harvest pathogen of many deciduous fruits worldwide. It is the etiological agent of the blue mold disease of pome fruits and is responsible for significant losses during post-harvest handling and storage of these commodities. As an infection strategy, *P. expansum* enhances its virulence by locally modulating the host pH through the production of organic acids, mainly citric and gluconic, and the efflux of H^+^ as a result of NH^4+^ utilization. The accumulation of these chemical species in the decaying fruit tissue leads to a reduction of pH by 0.5 to 1.0 units, which fosters the activity of polygalacturonase enzymes and the transcription of genes involved in fungal virulence (Prusky and Yakoby, 2003; Hadas et al., 2007; Prusky et al., 2007; Barad, Espeso et al., 2016). In agreement with these findings, it has been reported that *P. expansum* virulence and disease severity are much higher in apple cultivars characterized by low pH of the fruit tissue, or in fruits treated with citric acid (Marín et al., 2006; Kumar et al., 2018). Conversely, local alkalinization with sodium bicarbonate (NaHCO_3_) reduces decay development (Prusky et al., 2007). Furthermore, during the host tissue attack, *P. expansum* produces a large number of secondary metabolites, with patulin (PAT) being the most represented (Andersen et al., 2004). Patulin is a dangerous mycotoxin that contaminates stored fruits and derived products, representing a serious health hazard, especially for children. It has shown to be genotoxic on human cells *in vitro* (Glaser and Stopper, 2012; Pinedo et al., 2018) and possesses tumorigenic activity on mouse skin cells (Wright, 2015). Patulin can induce ROS-dependent damage by rapidly reacting with Glutathione, the depletion of which perturbates the cell redox homeostasis (Castoria et al., 2011; Ianiri et al., 2016). Therefore, maximum tolerable contamination levels of PAT have been set up (Commission Regulation, EC 1881/2006, 2006). The level of PAT synthesis varies considerably in different fungal strains and its biosynthesis and accumulation are influenced by environmental factors. Types of carbon sources and their concentration strongly influence PAT production by *P. expansum*, with glucose and glucose-containing sugars facilitating its production, while higher sugar concentrations reducing its accumulation (Barad et al., 2012; Prusky et al., 2014). Patulin production is also related to nitrogen availability, with organic nitrogen inducing higher PAT production than inorganic sources. In particular, ammonium sulfate was found as the most unfavorable nitrogen source for PAT biosynthesis (Rollins and Gaucher, 1994; Miyara et al., 2010; Zong et al., 2015). Another relevant factor affecting PAT biosynthesis is the extracellular pH. For the majority of *P. expansum* strains, acidic conditions promote higher PAT production than alkaline environments (Jimdjio et al., 2021). Nevertheless, the optimum of PAT biosynthesis is achieved in the pH range 4.0–5.0, while its production decreases at higher and lower pH values (Zong et al., 2015). Accordingly, PAT accumulation also depends on fruit cultivars and their degree of ripeness. For example, the tissue acidity of some apple varieties, such as Granny Smith (pH 2.90), allows lower PAT accumulation than less acidic varieties like Golden Delicious (pH 3.30; McCallum et al., 2002; Marín et al., 2006; Morales et al., 2007; Snini et al., 2016). Moreover, during apple maturation, the reduction of malic acid content and the decreasing fruit firmness increase susceptibility to *P. expansum* infection and PAT accumulation (Kumar et al., 2017).

In recent years, several studies evaluated the effect of control measures of mycotoxigenic fungal pathogens on mycotoxin biosynthesis. The effect of antifungal compounds on PAT biosynthesis is not unique because some of these induce downregulations of genes of the PAT biosynthetic pathway (Wang et al., 2018; Martínez-Culebras et al., 2021), while others (Delgado et al., 2022) stimulate its production and accumulation, especially if one considers the lower fungal biomass that develops in the decaying host tissue. Regarding the effect of microbial competitors of mycotoxigenic fungal pathogens on PAT biosynthesis and accumulation, such as yeasts acting as biocontrol agents (BCAs; De Curtis et al., 2004), a few studies report a reduction both *in vitro* and *in vivo* (i.e., in the fruit; Kabak et al., 2007; Castoria et al., 2008; Mahunu et al., 2016; Zheng et al., 2017; Nešić et al., 2021). The possible mechanisms involved are prevention of PAT contamination and detoxification. The prevention of PAT contamination is mainly based on controlling *P. expansum* through the reduction of conidial germination, germ tube elongation and inhibition of fungal growth, with a consequent decrease of PAT biosynthesis and accumulation (Yu et al., 2020). Conversely, PAT degradation consists of enzymatic degradation operated by several bacterial and yeast species leading to the formation of the end-products desoxypatulinic acid, (Z)-ascladiol, and hydroascladiol (Castoria et al., 2011; Ianiri et al., 2013; Mahunu, 2017; Tannous et al., 2017; Wei et al., 2020; Awuchi et al., 2021; Wang et al., 2022). Other studies show that some yeast species are also able to decrease PAT concentration through its adsorption to the cell wall (Caridi, 2007; McCormick, 2013; Nešić et al., 2021). However, the impact of these mechanisms on the decrease in PAT contamination *in vivo* has not yet been well understood. On the other hand, recent studies report that some BCAs, such as the basidiomycete yeasts *Rhodotorula mucilaginosa* strain 3617 and *R. kratochvilovae* strain LS11 increase the specific rate of PAT biosynthesis in *P. expansum* both *in vitro* and *in vivo*; however, the fungal growth, disease incidence and severity, and overall PAT accumulation were drastically reduced (Zheng et al., 2017).

In the present study, the effect of the interaction between the biocontrol yeast *P. terrestris* strain LS28 and *P. expansum* on environmental pH and PAT biosynthesis were investigates, with the aim of clarifying the factor(s) that influence the toxigenic activity and virulence of the fungal pathogen and the protection exerted by the BCA.

## Materials and methods

### Strains and growth conditions

The microorganisms used in this study were the mycotoxigenic postharvest pathogen *P. expansum* strain 7015, deposited in the “Toxigenic Fungi Culture Collection” of the Institute of Sciences of Food Production of the National Research Council (ISPA CNR—Bari, Italy), and the basidiomycetous yeast *P. terrestris* strain LS28, already known as a post-harvest biocontrol agent (Lima et al., 1999; Kheireddine et al., 2021). The fungal inoculum was prepared by culturing *P. expansum* on Potato Dextrose Agar medium (PDA) for 10 days at 25°C in the dark. The surface of the fungal culture was rinsed off with 10 ml of sterile distilled water containing 0.005% (v/v) Tween 80, and the concentration of the resulting conidial suspension was adjusted with a hemocytometer, as required for subsequent experiments. The BCA *P. terrestris* LS28 was routinely cultured in YPD medium (yeast extract at 10 g L^−1^, peptone at 20 g L^−1^, dextrose at 20 g L^−1^, and agar at 20 g L^−1^ for solid medium) at 28°C in shaking cultures at 150 rpm. Following incubation, cultures were centrifuged at 7,000 rpm for 5 min, washed twice with sterile distilled water and determination of cell concentration was performed using a hemocytometer.

### Measurements of pH

The effect of *in vitro* microbial interactions on pH trend and PAT accumulation was evaluated in apple mimicking medium (AMM), which was prepared by boiling 200 g of Golden Delicious apples for 30 min and filtration through sterile gauze to remove solid parts. The liquid volume was adjusted to 1 L that was autoclaved at 121°C for 20 min. For solid AMM, 18 g L^−1^ of agar was added.

To evaluate the role of the pH on PAT biosynthesis, pH of AMM was adjusted to 3.0, 4.0, 5.0, and 6.0 by using a buffer solution consisting of citric acid (0.1 mol L^−1^) and disodium phosphate Na_2_HPO_4_ (0.2 mol L^−1^), as reported by Furné et al. (2008). The *in vitro* experiments in liquid and solid AMM were carried out by inoculating *P. terrestris* at a concentration of 10^6^ cells ml^−1^ and *P. expansum* at a concentration of 10^3^ conidia ml^−1^. Cultures were incubated in rotary shakers at 150 rpm and 25°C for 7 days.

*P. expansum* conidia were washed twice with sterile distilled water and inoculated in 250 ml of different fresh media at a final concentration of 10^3^ conidia ml^−1^. Subsequently, 10^6^ cells ml^−1^ of *P. terrestris* LS28 were added to *P. expansum*-inoculated medium, and the culture was incubated at 25°C in agitation at 150 rpm. Cultures containing either *P. expansum* or LS28 alone were used as controls. To record the pH dynamics in liquid AMM, aliquots of the culture supernatant were withdrawn and subjected to pH measurement every 6 h using a pH-meter. Experiments were performed three times with three replicates for each experiment.

For a visual evaluation of the pH changes during microbial interactions, a 5-μl drop of conidial suspension of *P. expansum* at 10^8^ ml^−1^ was spotted onto 9-cm ⌀ Petri dishes containing 20 ml of agarized AMM and allowed to dry. Subsequently, 20 μl of suspension of LS28 at a concentration 10^8^ cells ml^−1^ were spotted 3 cm distant from the fungal spot and allowed to dry. After 5 days of incubation at 25°C, plates were overlaid with a 0.833 μM solution of the pH indicators bromocresol green or purple. The two microorganisms grown individually were used as controls. Experiments were performed at least four times, with two replicates in each experiment.

*In-vivo* assessment of pH trend was carried out on Golden Delicious apples purchased from a local market (Campobasso, Italy). The average values of fruit firmness and soluble solid concentrations were 3.4 kg and 14° Brix, respectively. Experiments included the time-course assessment of mesocarp pH and biocontrol assays in which LS28 was challenged by *P. expansum*. In the biocontrol experiments, LS28 was grown overnight in liquid YPD medium, the yeast cell suspension was washed twice with sterile distilled water and adjusted to concentrations of 5 × 10^6^ cells ml^−1^ (sub-optimal) and 10^8^ cells ml^−1^ (optimal). Apples were superficially sterilized by 1 min immersion in a sodium hypochlorite solution [1% (v/v) of active chlorine], rinsed twice with sterile distilled water and dried at room temperature. Four wounds (3 mm wide × 3 mm deep) were made on each fruit around the petiole using a cork borer. Immediately after wounding, 30 μl of the suboptimal or optimal LS28 cellular suspension was inoculated into each apple wound. After 30 min, 15 μl of a *P. expansum* suspension 2 × 10^4^ conidia ml^−1^ was added into the same artificial wounds. Mesocarp pH was measured at 0, 6, 12, 24, 48, 72, 96, 120, 144, and 176 h by placing a micro pH electrode directly on the exposed inoculated tissues, as previously described by Prusky and Yakoby (2003). For each treatment and each time point, measurements were repeated three times on 3 different fruits.

### Patulin extraction and HPLC quantification

The mycotoxigenic activity of *P. expansum* strain 7015, alone or co-incubated with the BCA *P. terrestris* LS28, was evaluated both *in vitro* and *in vivo*.

*In vitro* quantification of PAT was performed after 7 days of incubation in liquid AMM inoculated with the fungal pathogen as previously described. At the same time point (after 7 days of incubation), 20 ml of each culture was withdrawn and centrifuged at 6,000 rpm for 20 min: the pellet was heated at 65°C for 2 days to obtain the mycelial dry weight, while the supernatants were filtered with cellulose nitrate 0.45 μm filters and frozen at −20°C for successive mycotoxin quantification. Patulin was extracted from each supernatant with the same volumes of ethyl acetate for three times, according to AOAC (2000). The collected ethyl acetate (60 ml for each sample) was cleaned up with sodium carbonate solution (1.5% Na_2_CO_3_), dried with anhydrous sodium sulfate (Na_2_SO_4_), and then evaporated using a rotavapor system at 40°C. The dry residue was redissolved in 0.5 ml of mobile phase [H_2_O (acidified with 1% acetic acid) and methanol 95:5 (v/v)] and used for high-performance liquid chromatography (HPLC) determination as described below. For each treatment, three biological replicates were carried out.

Analyses of *in vitro* PAT production by *P. expansum* co-incubated with *P. terrestris* LS28 was carried out in solid AMM. To this aim, dual culture plates were set up as described above with some modifications. Briefly, a sterile cellophane paper was placed on top of the agar AMM medium, then the two microorganisms were spotted 3 cm apart as described in Section “Measurements of pH” and incubated for 10 days at 25°C in the dark. Afterwards, the cellophane with the fungal and yeast biomass was removed from the Petri dishes. The agarized AMM of each plate was finely shredded with a scalpel, transferred into a glass tube containing 20 ml of acidified distilled water, then PAT was extracted from this suspension three times with 15 ml of ethyl acetate (acidified with HCl). The solvent layer (45 ml) was dried as described above and redissolved in 0.5 ml of H_2_O (acidified with 1% acetic acid) and methanol 95:5 (v/v) for HPLC analysis. The experiments were repeated three times.

*In vivo* PAT production by *P. expansum* in rotting apple tissues, alone or co-inoculated with the BCA LS28, was assessed as previously described (Zheng et al., 2017) with slight modifications. Samples of rotting apple tissue for PAT quantification were collected 7 days after inoculation. Rotting tissues of the same technical replicate plus 1 cm of surrounding healthy tissue were withdrawn with a sterile scalpel, pooled, and stored at −80°C. To increase efficacy of extraction of both PAT and gDNA extraction (the latter for fungal biomass measurement described in Section “Measurement of *P. expansum* Biomass in Wounded Apple Tissue”), all the collected samples were lyophilized. Subsequently, 1 g of the lyophilized sample was grounded with a pestle, re-suspended in 5 ml of sterile distilled water, and PAT was extracted three times, each time with 5 ml of acidified ethyl acetate. The collected ethyl acetate layers (15 ml for each sample) were dried and redissolved in 0.5 ml of mobile phase (1% acetic acid solution and methanol 95:5, v/v), as described above. The samples were centrifuged, filtered through a 0.22 μm filter, and 20 μl were injected into an HPLC apparatus.

In all cases, HPLC analysis was carried out with a Dionex (Sunnyvale, CA, United States) analytical system consisting of a P680 solvent delivery system, and a 20 μl injector loop (Rheodyne, Cotati) was used for PAT determination. Detection was performed by measuring the absorbance of UV light at 276 nm by means of a UVD170 detector (Dionex, Sunnyvale, CA, United States) connected to a data integration system (Dionex Chromeleon, version 6.6). A Zorbax analytical column (SBC18 250 mm × 4.6 mm, 5 μm, Agilent Technologies) was used. The mobile phase, H_2_O (acidified with 1% acetic acid) with methanol 95:5 (v/v), was used with a flow rate of 1 ml/min and isocratic mode. Detection was performed by measuring the absorbance of UV light at 276 nm. Prior to sample analysis, a calibration curve was drawn by injecting triplicates of PAT standards at different concentrations (0, 4, 25, 50, 75, and 100 ppm). The standard solutions were prepared from a stock solution of 2,000 μg ml^−1^ of pure PAT (Sigma-Aldrich) solubilized in acidified ethyl acetate. The value of *R*^2^ was higher than 0.99. Each experiment consisted of three replicates per time point and each replicate consisted of three apples with four wounds each. Rotting wounds, collected from each replicate were pooled before extraction. Chromatographic analysis was performed in duplicates. Data from the three experiments, which were expressed as μg/ml of PAT, were similar as resulting from ANOVA analysis and were pooled. Bars in the figure represent mean values ± standard deviations.

### Measurement of *Penicillium expansum* biomass in wounded apple tissue

Real-time quantitative PCR (RT qPCR) was used to quantify the biomass of *P. expansum* strain 7015 grown in wounded apple tissues when inoculated alone or co-inoculated with the BCA *P. terrestris* LS28. To this aim, total DNA extraction and purification were performed as described by Zheng et al. (2017) from 0.5 grams of the same samples of lyophilized apple tissue that were withdrawn for PAT analyses (see above). RT qPCR was performed by using specific primer pairs designed on the *PatF* gene, patF_F (ATGAAATCCTCCCTGTGGGTTAGT) and patF_R (GAAGGATAATTTCCGGGGTAGTCATT), reported by Tannous et al. (2015). Reaction mixtures contained 10.0 μl of SYBR Premix Ex TaqTM (Takara), 0.4 μl of each primer (20 mM) and 2 μl of total DNA extracted from the apple samples in a final volume of 20 μl. Two simultaneous replicated amplifications were carried out. Amplification reactions were performed in 96-well microtiter plates (Twin.tec PCR plate, Eppendorf) and a thermal cycler apparatus (Mastercycles EP gradient S, Eppendorf) by using the following cycling protocol: an initial denaturation step (30 s at 95°C) followed by 40 cycles of 5 s at 95°C, 20 s at 55°C, and 20 s at 72°C. Afterwards, a melting curve program was run in which measurements were made at 0.5°C T increments every 10 s within a range of 60–95°C. The DNA concentration of each sample was extrapolated from a standard curve which was developed by plotting the logarithm of known concentrations (10-fold dilution series from 10 ng to 1 pg., 25 μl reaction) of *P. expansum* genomic DNA.

### Determination of the specific mycotoxigenic activity *in vitro* and *in vivo*

The specific rate of patulin biosynthesis by *P. expansum* strain 7015 in liquid AMM medium was calculated by referring the mean amount of patulin (μg) to the weight (g) of dry fungal mycelium or mL of cultural broth, depending on the experiment; in agarized AAM assays, the amount of patulin was referred to the respective fungal biomass expressed as cm of fungal radial growth. In *in vivo* experiments, the specific mycotoxigenic activity by *P. expansum* was expressed as ng patulin per μg of fungal DNA, as already reported in the work by Zheng et al. (2017).

### Statistical analysis

Data were submitted to factorial analysis of variance (one-way ANOVA) followed by Uncorrected Fisher’s LSD comparison test. Differences were considered statistically significant for *p* < 0.05. The percentages of infected wounds were converted into Bliss angular values (arcsine √%) before statistical analysis. The comparisons between two groups were carried out using a two-tailed unpaired Student’s *t*-test. Statistical analyses were performed using the GraphPad software 8.

## Results

### Effect of *Papiliotrema terrestris* on patulin accumulation by *Penicillium expansum* and mycotoxigenic activity *in vivo*

**Figure 1:**
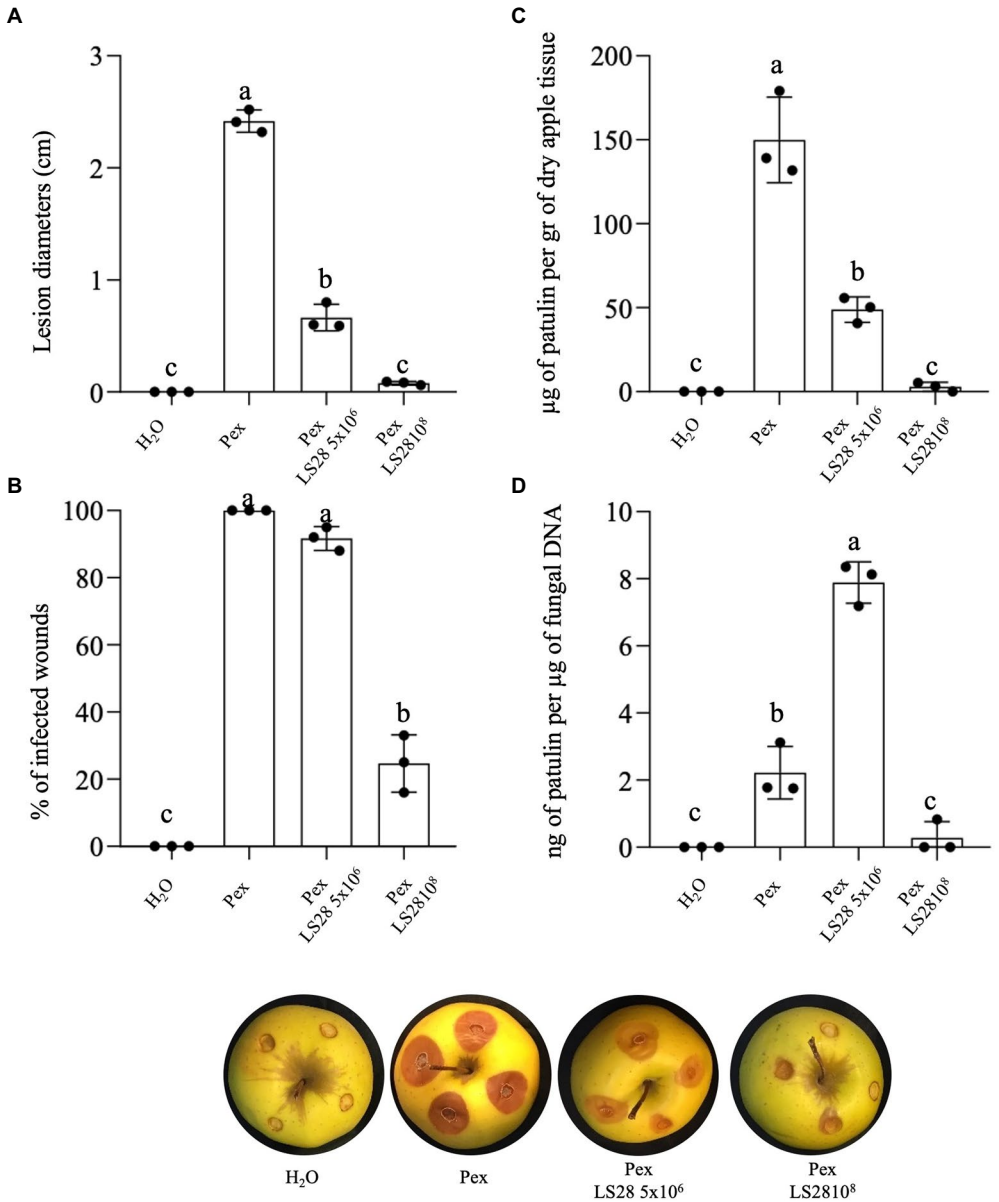
Effect of optimal (1 × 10^8^ CFU ml^−1^) and suboptimal (5 × 10^6^ CFU ml^−1^) concentrations of the biocontrol yeast *Papiliotrema terrestris* LS28 after 7 days of incubation on **(A)** lesion diameter; **(B)** percentage of infected wounds; **(C)** patulin accumulation; **(D)** specific mycotoxigenic activity, in apples inoculated with *P. expansum* strain 7015 (Pex). Columns with common letters are not significantly different according to one-way ANOVA followed by Uncorrected Fisher’s LSD comparison test (*p* < 0.05). Data shown are the mean values ± SD from three independent experiments (black dots).

The effects of *P. terrestris* LS28 was evaluated *in vivo* on green blue mold disease development and toxigenic activity of *P. expansum* strain 7015 by analyzing the percentage of infected wounds and PAT biosynthesis during the tri-trophic interaction between the host (apple fruit), the pathogen, and the biocontrol agent. The BCA *P. terrestris* LS28 was applied at optimal and sub-optimal cell concentrations. As expected, and in agreement with previous data (Lima et al., 1998, 1999, 2005; Castoria et al., 2003, 2021) after 7 days of incubation the application of the BCA at the optimal cell concentration results in a significantly lower percentage of wounds infected by *P. expansum* as compared to the fungal pathogen applied alone (Figures 1A,B). Furthermore, the high antagonistic activity of *P. terrestris* LS28 at the optimal cell concentration is associated to a substantial reduction of PAT accumulation in rotting tissues as compared to the positive control, both in terms of μg of PAT per gram of lyophilized apple tissues (Figure 1C), and ng of PAT per μg of fungal DNA (Figure 1D). In the control treatment, *P. expansum* inoculated alone is able to produce approximately 150 μg of PAT per gram of dry apple tissue (Figure 1C), and 2 ng of PAT per μg of fungal DNA (Figure 1D). Conversely, the (lower) suboptimal concentration of the BCA LS28 causes a significant reduction in the disease severity induced by *P. expansum*, although at a lesser extent than the optimal BCA concentration (Figure 1A) but provided almost no protection from *P. expansum* infection (Figure 1B). Moreover, when considering the amount of PAT (μg) per gram of dry apple tissue, the lower protection operated by the BCA resulted in a higher level of PAT contamination as compared to the treatment with the optimal yeast concentration, although lower than the positive control (Figure 1C). Strikingly, the suboptimal concentration of the BCA LS28 strongly stimulates PAT biosynthesis, as shown by the specific toxigenic activity of *P. expansum* (~8 ng of PAT per μg of fungal DNA; Figure 1D), despite the overall contamination level being lower than the control (Figure 1C). In particular, the co-incubation of the pathogen with the suboptimal concentration of *P. terrestris* cells leads to an approximately fourfold increase of the specific mycotoxigenic activity of *P. expansum* as compared to the positive control (*P. expansum* alone), with 7.8 and 1.8 ng of PAT per μg of fungal DNA, respectively.

### *In vitro* the BCA stimulates patulin accumulation

**Figure 2:**
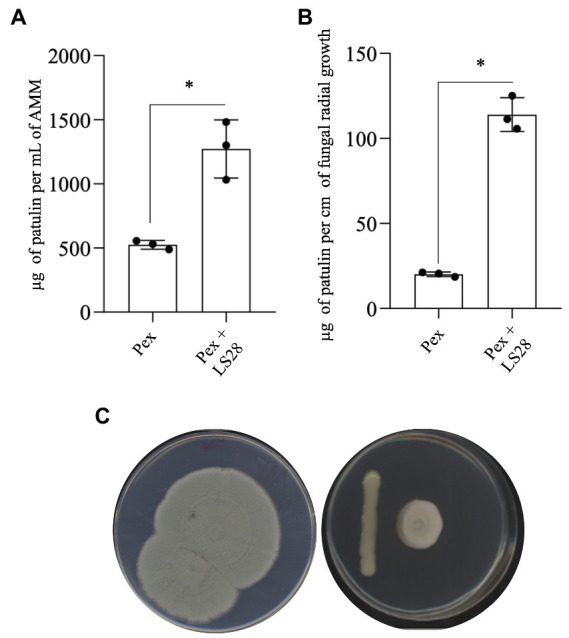
Patulin quantification in dual cultures of *Penicillium expansum* strain 7015 (Pex) and the biocontrol agent *Papiliotrema terrestris* LS28 in **(A)** liquid AMM (μg patulin ml^−1^ of medium) and **(B)** agarized AMM (μg patulin cm^−1^ of fungal radial growth). Patulin was extracted and quantified 7 days after the inoculation. Data are presented as mean values ± SD from three independent experiments (black dots). *p < 0.05 vs. Pex according to two-tailed, unpaired Student’s *t*-test.

Patulin biosynthesis by *P. expansum* during the interaction with the biocontrol agent *P. terrestris* LS28 was evaluated *in vitro* co-culturing the two microorganisms in liquid and solid AMM. In liquid assays, after 7 days, the fungal pathogen alone is able to accumulate 525 μg of PAT per ml^−1^ of broth. Co-incubation with LS28 strongly stimulates mycotoxin biosynthesis, with a PAT content of 1,270 μg ml^−1^ of cultural broth (Figure 2A). A similar approach was carried out on solid AMM. The dual culture plates were kept in incubation until the fungal colony covered the entire Petri dish (Figure 2C) in the control condition (fungal alone); at this time point, PAT was extracted and quantified, and the mycotoxin content was expressed as μg per cm of radial growth (Figure 2B). As in the case of the experiments in liquid medium, PAT biosynthesis by *P. expansum* is much higher in the presence of the BCA (113.9 μg per cm of fungal radial growth) as compared to the fungal pathogen alone (20.5 μg per cm of fungal radial growth).

### *Penicillium expansum* toxigenic activity is pH-dependent *in vitro*

**Figure 3:**
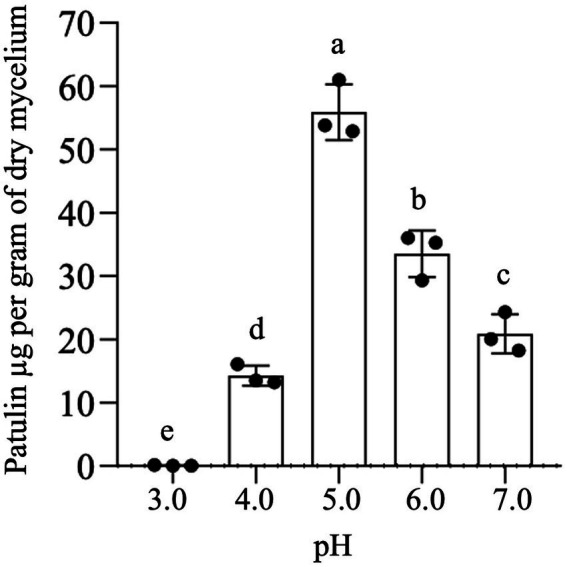
*In vitro* patulin production at fixed pH values. Patulin was extracted from a 7-day-old culture of *Penicillium expansum* strain 7015 in liquid AAM in which the pH was kept unvaried at fixed values. Data shown are the mean values ± SD from three independent experiments (black dots). Columns with common letters are not significantly different according to one-way ANOVA followed by uncorrected Fisher’s LSD comparison test (*p* < 0.05).

To elucidate the effect of extracellular pH on the specific mycotoxigenic activity of *P. expansum* strain 7015, the pathogen was cultured in liquid AAM at pH values ranging from 3 to 7. After 7 days of the inoculation, PAT content in the cultural broth was determined and expressed in relation to the fungal dry biomass of each condition. As reported in Figure 3, PAT production in AMM is strongly related to pH value. In particular, the highest specific mycotoxigenic activity is achieved at pH 5, whereas it decreases at both higher and lower pH values, being the lowest at pH 3.

### Influence of *Papiliotrema terrestris* on *Penicillium expansum*-induced acidification *in vitro*

As already known for *P. expansum* (Prusky and Yakoby, 2003; Hadas et al., 2007; Prusky et al., 2007; Barad, Espeso et al., 2016; Barad, Sionov et al., 2016; Tannous et al., 2018), and as confirmed for the strain 7015 used in this study (Figure 3; see above), PAT biosynthesis is strongly influenced by environmental pH.

**Figure 4:**
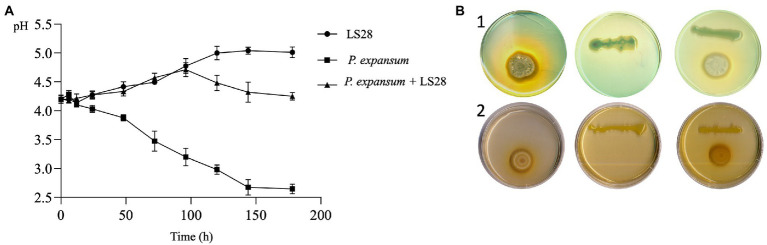
**(A)** *In vitro* extracellular pH trend of *P. expansum* co-inoculated with LS28 in liquid AMM. pH was measured at 0, 6, 12, 24, 48, 72, 96, 120, 144, and 178 h after co-inoculation. Data shown are the mean values ± SD from three independent experiments. **(B)** Representative pictures showing *P. terrestris* strain LS28 and *P. expansum* strain 7015 grown alone or in dual cocultures for 7 days in AMM plates containing the pH indicators bromocresol green (yellow, pH < 3.8; blue, pH > 5.4) or purple (yellow, pH < 5.2; purple, pH > 6.8). In the plates amended with the pH indicator bromocresol green (b-1), the extracellular acidification produced by *P. expansum* growth is visible as a clear halo, while this pH changing is counteracted by the biocontrol yeast; No color changes occur in the plates with the pH indicator bromocresol purple (b-2).

To investigate the involvement of this mechanism in the interaction between *P. terrestris* strain LS28 and the fungal pathogen, the extracellular pH was monitored over time *in vitro*, both in solid and liquid AAM media, either with the microorganisms grown alone or in co-incubation. As shown in Figure 4A, *P. expansum* alone quickly acidifies the extracellular environmental pH from 4.2 to 3.6 at 48 h. The tissue acidification driven by the fungal proliferation gradually continues until it reached pH 2.6 at 144 h of incubation. Conversely, after the same time interval, *P. terrestris* LS28 increases by approximately 0.8 unit (pH 5.0) the extracellular pH compared to the first time point (pH 4.2). Interestingly, in dual culture the biocontrol agent induces an increase of the extracellular pH from 4.2 to 4.5 after 96 h of incubation, thus apparently counteracting the rapid acidification caused by *P. expansum* (Figure 4A). At the latter time points, the pH slightly decreases to reach a value of 4.2 at 144 h of incubation.

The effect of *P. terrestris* LS28 on extracellular pH was also investigated on agar AMM by using the pH indicators green and purple bromocresol, which allowed to visualize pH variations during the dual interaction. The pictures in Figure 4B clearly show that after 7 days from the inoculation, *P. expansum* 7015 strongly acidifies the agar medium by producing a yellow halo surrounding the fungal colony in the presence of both purple and green bromocresol. Conversely, *P. terrestris* LS28 grown alone on AMM leads to a slight color change in the presence of bromocresol purple, while no color variations are observed with bromocresol green. In the dual culture, the biocontrol agent LS28 apparently counteracts the acidification produced by the pathogen so that no color change occurs in the presence of bromocresol green.

### *In vivo* influence of *Papiliotrema terrestris* on *Penicillium expansum*-induced acidification of apple wounds

**Figure 5:**
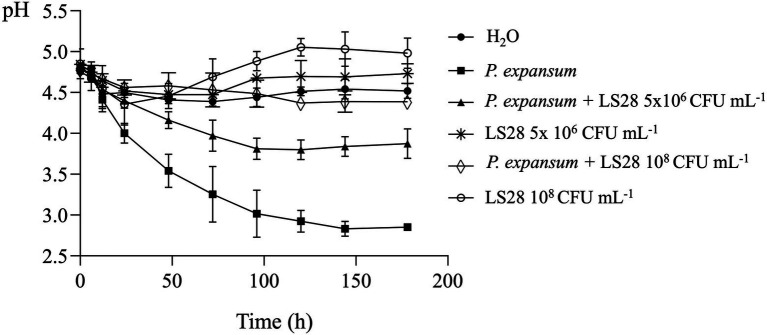
*In vivo* extracellular pH trend in apple wounds inoculated with optimal (10^8^ CFU ml^−1^) and suboptimal (5 × 10^6^ CFU ml^−1^) concentrations of the biocontrol yeast *Papiliotrema terrestris* LS28 and with the fungal pathogen *Penicillium expansum* strain 7015. pH was measured at 0, 6, 12, 24, 48, 72, 96, 120, 144, and 178 h after inoculation. Data shown are the mean values ± SD from three independent experiments.

For assessing the *in vivo* pH trend, *P. expansum* and two different concentrations (optimal and suboptimal) of the biocontrol yeast were inoculated in apple wounds, and wound pH monitored over time (Figure 5). In *P. expansum*-inoculated wounds, as expected there was a rapid acidification with pH value of 3.5 at 48 h of incubation; pH values further decrease to reach 2.8 at 168 h of incubation. *P. terrestris* LS28 alone causes only slight pH changes in apple wounds, which display a trend like that of uninoculated healthy wounds: the inoculum with lower suboptimal yeast cell concentrations induces a slight pH increase during incubation, whereas the higher optimal concentration of the BCA induces a more pronounced alkalinization, with pH value of 5.0 after 168 h of incubation. As in the case of *in vitro* experiments (Figure 4), in apple wounds co-inoculated with LS28 and *P. expansum*, the BCA appears to counteract the acidification produced by the fungal pathogen with an efficacy that is correlated to the concentration of the yeast cells: the co-incubation of 5 × 10^6^ CFU ml^−1^ of LS28 and *P. expansum* produces a lower acidification than the pathogen alone, stabilizing the pH at 3.8 after 96 h from inoculation. Conversely, the optimal concentration of LS28 keeps the pH unchanged during the incubation with a trend similar to H_2_O-treated healthy wounds, in which the pH remains stable over time, ranging from pH 4.7 to 4.5 for the whole duration of the experiments.

## Discussion

The ability to perceive and adapt to the environment is a key aspect for which both eukaryotes and prokaryotes invest in terms of energy. Many cellular responses intimately depend on a plethora of external variables and, among these, pH emerges as a crucial parameter which all microorganisms influence and depend on (Kemmitt et al., 2006; Falkowski et al., 2008; Bethke et al., 2011; Lennon and Jones, 2011; Amend et al., 2013; Maguffin et al., 2015; Zhalnina et al., 2015). The pH is also a crucial factor in the infection process. Several fungal plant pathogens are able to finely change the pH in the infection site, thus achieving the optimal condition for the expression of virulence-related genes through a process driven by different modulators (Rodriguez-Moreno et al., 2018; Li et al., 2020; Laethem et al., 2021). *Penicillium expansum* is the causative agent of green-blue mold on stored pome fruits. This fungus secretes a combination of organic acids to acidify the infection site, thus modulating the expression of virulence-related genes and creating a favorable condition for the activity of polygalacturonases, enzymes that are responsible for cleavage and depolymerization of pectin, a major component of middle lamella. During its infection process *P. expansum* produces also the mycotoxin PAT, although its active role in pathogenesis is controversial (Prusky and Yakoby, 2003; Hadas et al., 2007; Prusky et al., 2007; Puel et al., 2010; Sanzani et al., 2012; Barad et al., 2013; Barad, Sionov et al., 2016; Li et al., 2020; Bartholomew et al., 2021). Although many aspects of PAT biosynthesis have been elucidated, to date little is known about the effect of pH modulation on PAT biosynthesis *in vivo*, i.e., on apple fruits infected by *P. expansum* (Moake et al., 2005; Jimdjio et al., 2021). Similarly, no reports exist on the effect of biocontrol agents on host tissue pH when they counteract the attack on fruits by *P. expansum*.

In the present study, the effect of an optimal and a suboptimal concentration of the biocontrol agent *P. terrestris* LS28 on disease incidence and severity, and on PAT biosynthesis in apples inoculated with *P. expansum* was initially investigated. In agreement with our previous results (Lima et al., 1998, 1999, 2005; Castoria et al., 2003, 2021) the optimal concentration of the BCA *P. terrestris* LS28 provides almost full protection of artificially infected apple wounds after 7 days, both in terms of disease severity and disease incidence (i.e., lesion diameters and percentage of infected wounds, respectively; Figures 1A,B). The biocontrol activity of *P. terrestris* LS28 also influences PAT accumulation by reducing the total amount of this mycotoxin in infected apples (Figure 1C). As expected, the lower concentration of the BCA results in a lower biocontrol activity, but determined an increase of the specific mycotoxigenic activity by *P. expansum* (Figure 1D), even if the overall contamination with PAT is reduced also in this case (Figure 1C). Moreover, the increased PAT production by *P. expansum* in the presence of LS28 also occurs in *in vitro* experiments performed both in liquid and solid media (Figure 2), especially when the amount of PAT was referred to a parameter mirroring the fungal biomass in the dual culture (i.e., fungal radial growth; Figure 2B). Data obtained from the above-mentioned *in vivo* and *in vitro* experiments are in agreement with those reported by Zheng et al. (2017) who investigated the effects of two biocontrol yeasts, *R. mucilaginosa* 3617 and *R. kratochvilovae* (syn. *Rhodosporidium kratochvilovae*) LS11, on green blue mold disease and PAT contamination caused by two different strains of *P. expansum* (PY and FS7) in artificially inoculated Fuji apples. While the BCAs significantly reduced the disease symptoms and lowered the development of fungal biomass, they both increased the specific rate of PAT production even though they reduced the total mycotoxin contamination. Similarly, Zhu et al. (2015) reported the effect of the marine yeast *R. paludigenum* on PAT accumulation in apples and pears. The occurrence and severity of apple and pear decay caused by *P. expansum* were significantly inhibited, but the yeast enhanced PAT accumulation as compared to the controls in infected fruits.

Next, we aimed to elucidate the mechanisms responsible for the increased specific mycotoxigenic activity by *P. expansum* in the presence of a suboptimal concentration of the biocontrol agent *P. terrestris* LS28. First, because it is known that PAT production by *P. expansum* is influenced by the pH (Zong et al., 2015), the *P. expansum* strain 7015 specific mycotoxigenic activity was determined in apple mimicking medium in which fixed pH values were kept constant over time. In agreement with Zong et al. (2015), PAT production is closely related to pH, with its biosynthesis occurring at pH between 4 and 7, and with the optimum at pH 5 (Figure 3). Following this observation, we speculated that the biocontrol agent *P. terrestris* LS28 might influence PAT production by *P. expansum* by modifying the pH of the media. To assess this hypothesis, *in vitro* experiments were performed by growing *P. terrestris* strain LS28 and *P. expansum* strain 7015 alone or co-incubated in both liquid and solid artificial apple medium and by monitoring the pH at determined time points. Our results clearly show that *P. terrestris* is able to counteract the strong acidification induced by *P. expansum*, with pH value remaining almost unchanged (4.2–4.5) for the whole duration of the experiments (Figure 4A). This buffering effect of *P. terrestris* against the extracellular acidification induced by the fungal pathogen is also observed *in vivo*, i.e., in apple wounds, and the pH increase induced by *P. terrestris* strain LS28 is proportional to the cellular concentration used, being more prominent when a high number of *P. terrestris* cells were used (Figure 5).

Remarkably, the pH increase determined by *P. terrestris* when coincubated with *P. expansum* falls within or close to the range in which the highest production of PAT by *P. expansum* is detected, clearly suggesting that the buffering effect exhibited by *P. terrestris* against the acidification induced by *P. expansum* accidentally is responsible for the increased mycotoxigenic activity of the fungus. It is likely that the increased PAT production by *P. expansum* during co-cultivation with other BCAs of the genus *Rhodosporidium* (Zhu et al., 2015; Zheng et al., 2017) might be also due to a modulation of the extracellular pH, although this parameter had not been investigated by the aformentioned authors. If this hypothesis is true, the mechanisms of extracellular pH modulation induced by the BCA might be a conserved feature of Basidiomycetous BCAs and its specific role in biocontrol acitivity should be studied in more details. In this regard, we have unpublished evidences that the addition of alkalinizing or acidifying compounds during microbial competition between *P. terrestris* LS28 and *P. expansum* in apple wounds leads to an increase or a decrease in the yeast biocontrol activity, respectively. Nevertheless, in these experiments the compounds that were used to modulate the apple wounds pH were also a source of nutrients both for the BCA and the fungus (e.g., ammonium sulphate, an acidifying compound that is also a source of nitrogen and sulfur), and as a consequence, it was difficult to exclusively determine the role of the pH modulation in the biocontrol activity of *P. terrestris* LS28 (data not shown). Therefore, our data are not conclusive and further studies are necessary to define the role of the extracellular pH buffering in the biocontrol activity of *P. terrestris* LS28.

It is interesting to note that the buffering activity displayed by *P. terrestris* LS28 is not triggered by the presence of the fungus, since there is a more pronounced pH increase where the BCA was inoculated alone, both *in vitro* and *in vivo* (Figures 4, 5). Although this mechanism is unknown, it can be speculated that the pH increase might be due to the production and secretion by the BCA of an alkaline compound, which then would counteract tissues acidification induced by certain plant pathogens as *P. expansum*. On the other hand, environmental alkalinization due to ammonia production is also a known virulence factor for some other plant pathogens such as *Colletotrichum gloeosporioides,* the cause of anthracnose fruit rot (Shnaiderman et al., 2013), and in this case the buffering activity of *P. terrestris* LS28 might be advantageous for the phytopathogenic fungus.

Besides pH modulation, as noted by Zheng et al. (2017), a contribution to the increase of mycotoxigenic activity by *P. expansum* could also be due to the nutritional stress encountered by this pathogen during competition for nutrients operated by the BCA, since the association between nutritional imbalance and the induction of secondary metabolism of *P. expansum* has recently been reported (Tannous et al., 2018).

## Conclusion

Our results show that the biocontrol agent *P. terrestris* LS28 is effective in controlling green blue mold caused by *P. expansum* and in extremely reducing PAT contamination in apple fruits. This reduction occurs even if this biocontrol yeast causes an increase of specific mycotoxigenic activity by the pathogen, as measured as the quantity of PAT per unit of fungal biomass. This increase seems to be associated to the alkalinization of the infection court operated by *P. terrestris* LS28, which keeps the pH value within the range in which *P. expansum* synthesizes the highest level of PAT. Moreover, the ability to counteract the *P. expansum*-driven acidification, a pivotal mechanism for the fungal attack to the host fruit, appears to be also associated to the protection of fruits, suggesting a potential novel biocontrol mechanism operated by *P. terrestris* LS28 to antagonize *P. expansum* infection. While further studies are necessary to validate this hypothesis, to our knowledge this is the first report on the influence of a biocontrol agent on pH of the infection court of a postharvest pathogen, and on the consequent effects on mycotoxin synthesis and the level of fruit protection.

## Data availability statement

The raw data supporting the conclusions of this article will be made available by the authors, without undue reservation.

## Author contributions

DP, GI, and RC: designed the experiment, analyzed results, wrote, and revised the manuscript. CM: provided suggestion for the experimental design, performed the experiments, analyzed results, and revised the manuscript. IN and PA: performed HPLC analyses, analyzed results, and revised the manuscript. GL and FD: analyzed results and revised the manuscript. All authors contributed to the article and approved the submitted version.

## Funding

GI and RC are supported by the PON AIM program (AIM1804798) Azione I.2 “Attrazione e Mobilità dei Ricercatori.”

## Conflict of interest

The authors declare that the research was conducted in the absence of any commercial or financial relationships that could be construed as a potential conflict of interest.

## Publisher’s note

All claims expressed in this article are solely those of the authors and do not necessarily represent those of their affiliated organizations, or those of the publisher, the editors and the reviewers. Any product that may be evaluated in this article, or claim that may be made by its manufacturer, is not guaranteed or endorsed by the publisher.
